# Supplementary figures and images for: Endosomal egress and intercellular transmission of hepatic ApoE-containing lipoproteins and its exploitation by the hepatitis C virus
Source: PLoS Pathog. 2023 Jul 28;19(7):e1011052. doi: 10.1371/journal.ppat.1011052 (PMC10411793; doi:10.1371/journal.ppat.1011052)

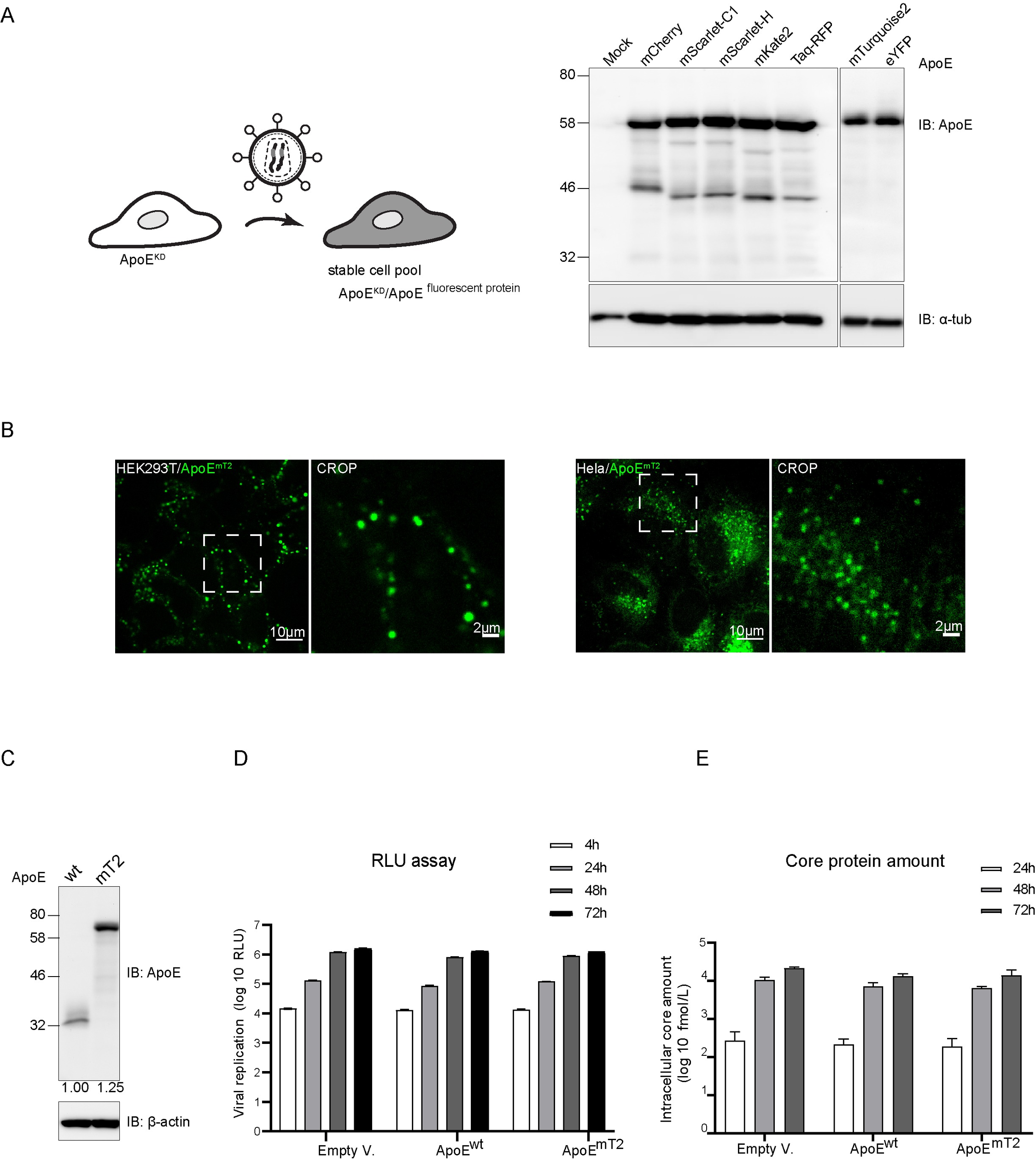

Supplement: S1 Fig — (A) Validation of ApoE tagging with various fluorophores and confirmation of expression. Huh7-Lunet cells with stable knockdown (KD) of ApoE were transduced with lentiviruses encoding different fluorescently tagged-ApoE variants. After selection for stable expression, lysates of given cell pools were analyzed by Western blot using an ApoE-specific antibody. α-tubulin served as a loading control. mScarlet-C1: wildtype mScarlet; mScarlet-H: photo-stable mScarlet (M164H) variant. (B) Subcellular distribution of ApoEmT2 in HEK293T (left) and Hela cells (right) stably expressing this protein after lentiviral transduction and selection. Cells were characterized by confocal microscopy. (C) Expression of ApoEwt (wt) and ApoEmT2 (mT2) in reconstituted Huh7-Lunet cells with stable depletion of endogenous ApoE (Huh7-Lunet/ApoE-KD cells) was examined by Western blot using an ApoE-specific antibody. β-actin served as a loading control. Level of ApoE protein expression relative to wt (set to 1) is given below the lanes. (D-E) HCV replication in Huh7-Lunet/ApoEmT2 cells. (D) Cells were transduced with either an empty vector (Empty V), or ApoEwt, or ApoEmT2, respectively, and selected for stable transgene expression. Cells were then electroporated with in vitro transcripts of the HCV Renilla luciferase (RLU)-reporter virus (JcR2a). HCV replication was determined at indicated time points by measuring RLU activities in cell lysates. (E) Amounts of core protein contained in cells from (D) at indicated time points were measured by chemiluminescence assay. Data are means of internal replicates from a representative experiment (n = 3). (TIF) [file ppat.1011052.s001.tif]

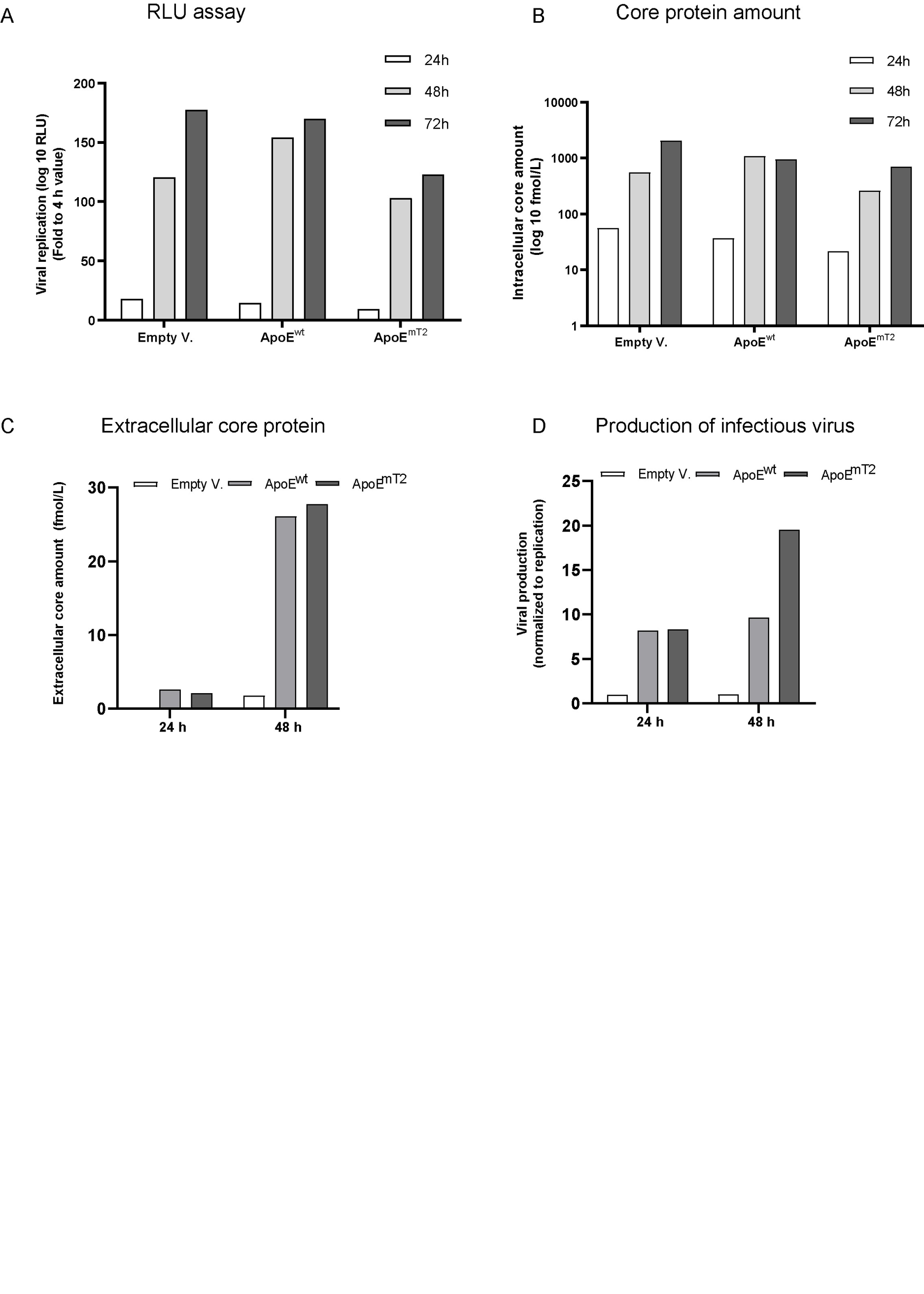

Supplement: S2 Fig — (A-B) HCV replication in HEK293T-miR122-ApoEmT2 cells. (A) Cells were transduced with either an empty vector (Empty V.), or wildtype ApoE (ApoEwt), or ApoEmT2, respectively, and electroporated with in vitro transcripts of the HCV Renilla luciferase (RLU)-reporter virus (JcR2a). HCV replication was determined at indicated time points by measuring RLU activities in cell lysates. RLU activities were normalized to the 4 h value to correct for the transfection efficiency. (B) Amounts of core protein contained in cells from (A) at indicated time points were measured by chemiluminescence assay. Data in both panels are means for a representative experiment (n = 2). (C-D) Production of infectious HCV in HEK293T-miR122-ApoEmT2 cells. (C) At 24 and 48 h post-electroporation, amounts of extracellular core protein present in supernatants of cells from (A) were determined by chemiluminescence assay. (D) Culture supernatants harvested at 24 and 48 h post-electroporation were used to inoculate naïve Huh7.5 cells and HCV replication therein was measured by quantifying RLU activity at 72 h after inoculation. Virus titers normalized to HCV RNA replication in transfected cells are shown. Data in both panels are means for a representative experiment (n = 2). (TIF) [file ppat.1011052.s002.tif]

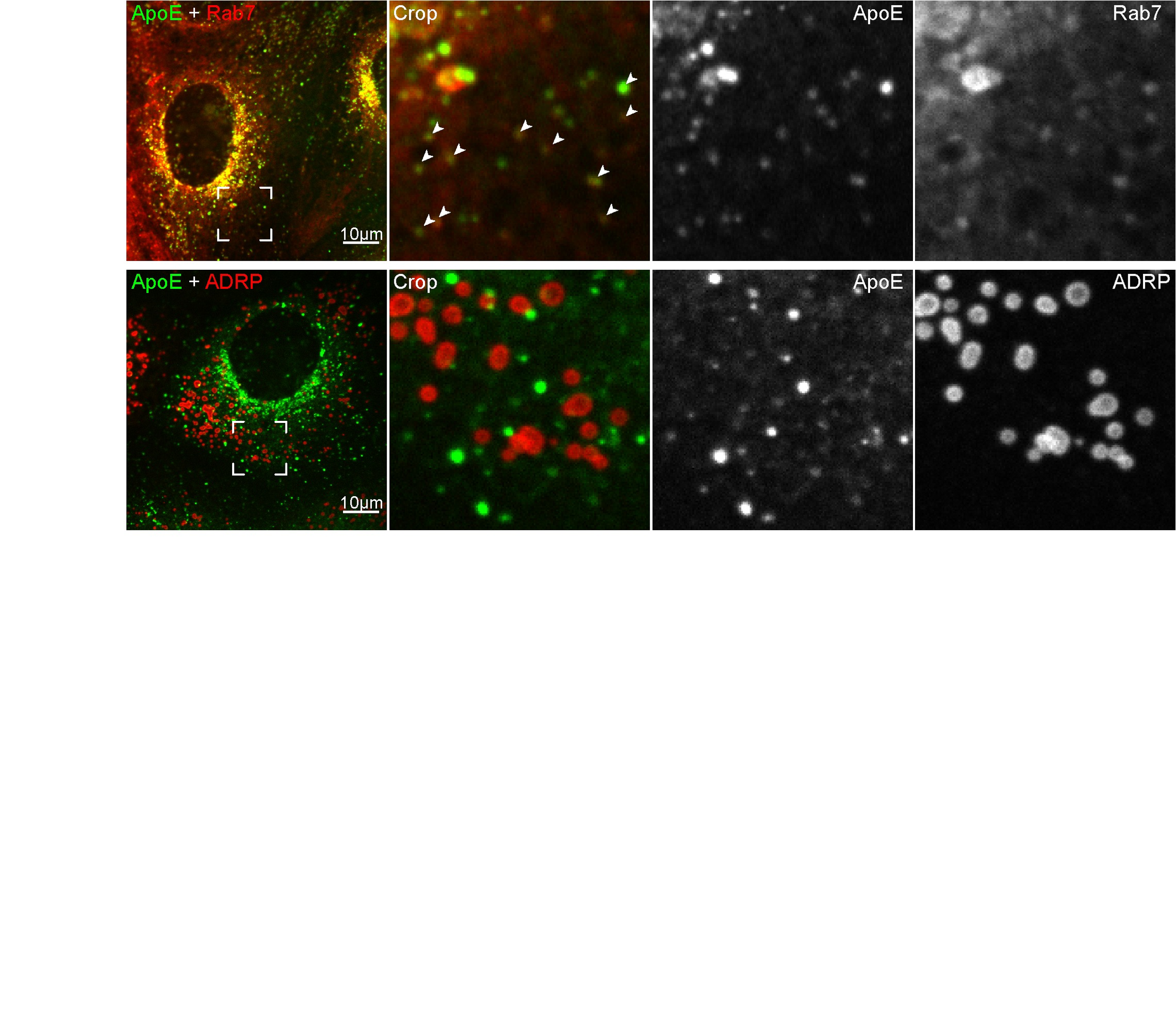

Supplement: S3 Fig — Huh7-Lunet/ApoEmT2 cells were transduced with lentiviruses encoding Rab7mCherry (upper panel) or ADRPmCherry (lower panel). Cells were fixed and analyzed by confocal microscopy. Boxed areas in the left panels are shown as enlarged views in the panels on the right of each row. Arrowheads point to ApoE-Rab7 positive signals. (TIF) [file ppat.1011052.s003.tif]

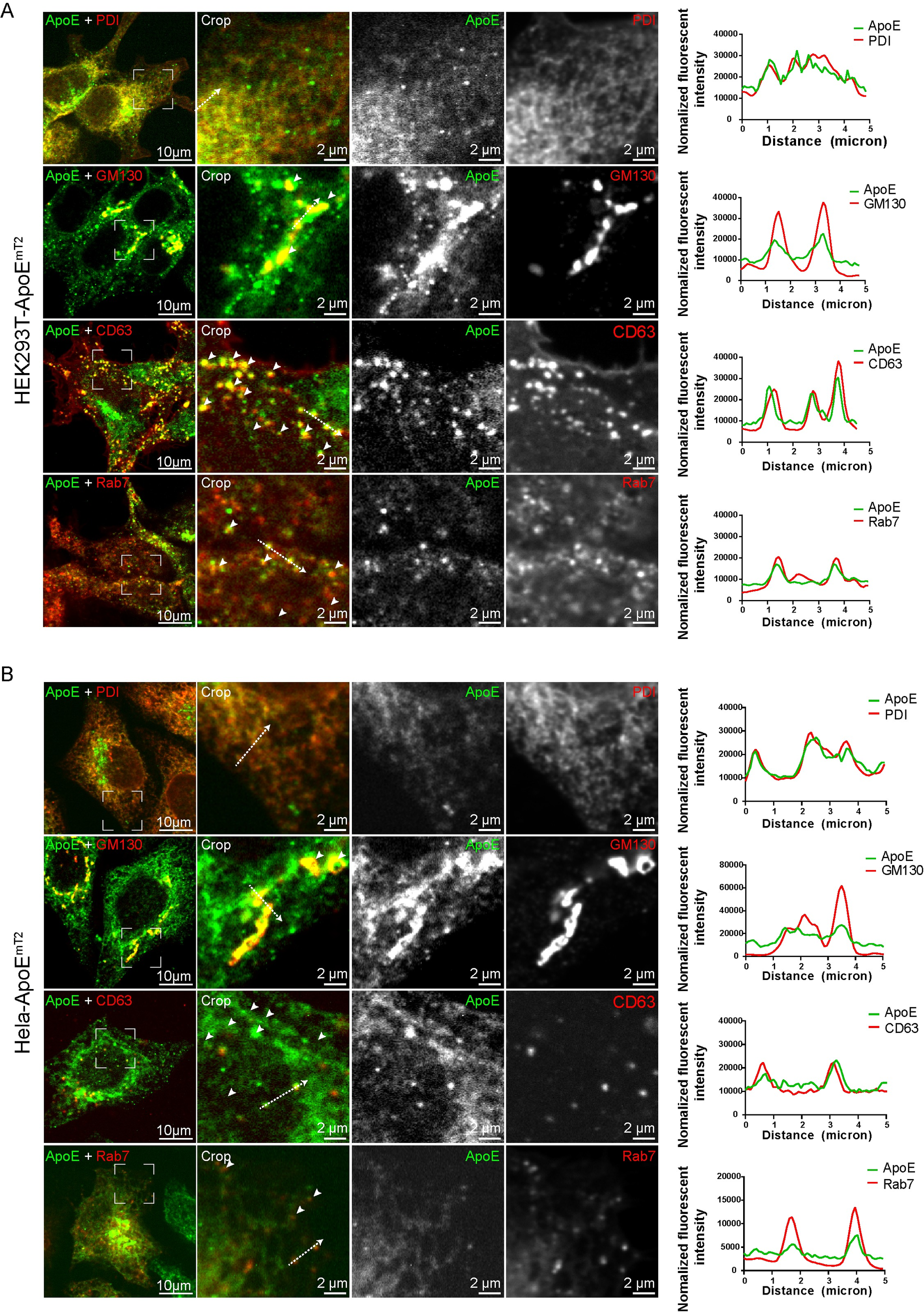

Supplement: S4 Fig — HEK293T/ApoEmT2 (A) and Hela/ApoEmT2 cells (B) were subjected to immunostaining of markers of the ER (PDI), Golgi (GM130), or transduced with lentiviruses encoding CD63mCherry or Rab7mCherry to label intraluminal vesicles/endosomes, and analyzed by confocal microscopy. Boxed areas in the left panels are shown as enlarged views in the panels on the right of each row. Profiles on the right of each panel were taken along the lines indicated with white arrows in cropped images. (TIF) [file ppat.1011052.s004.tif]

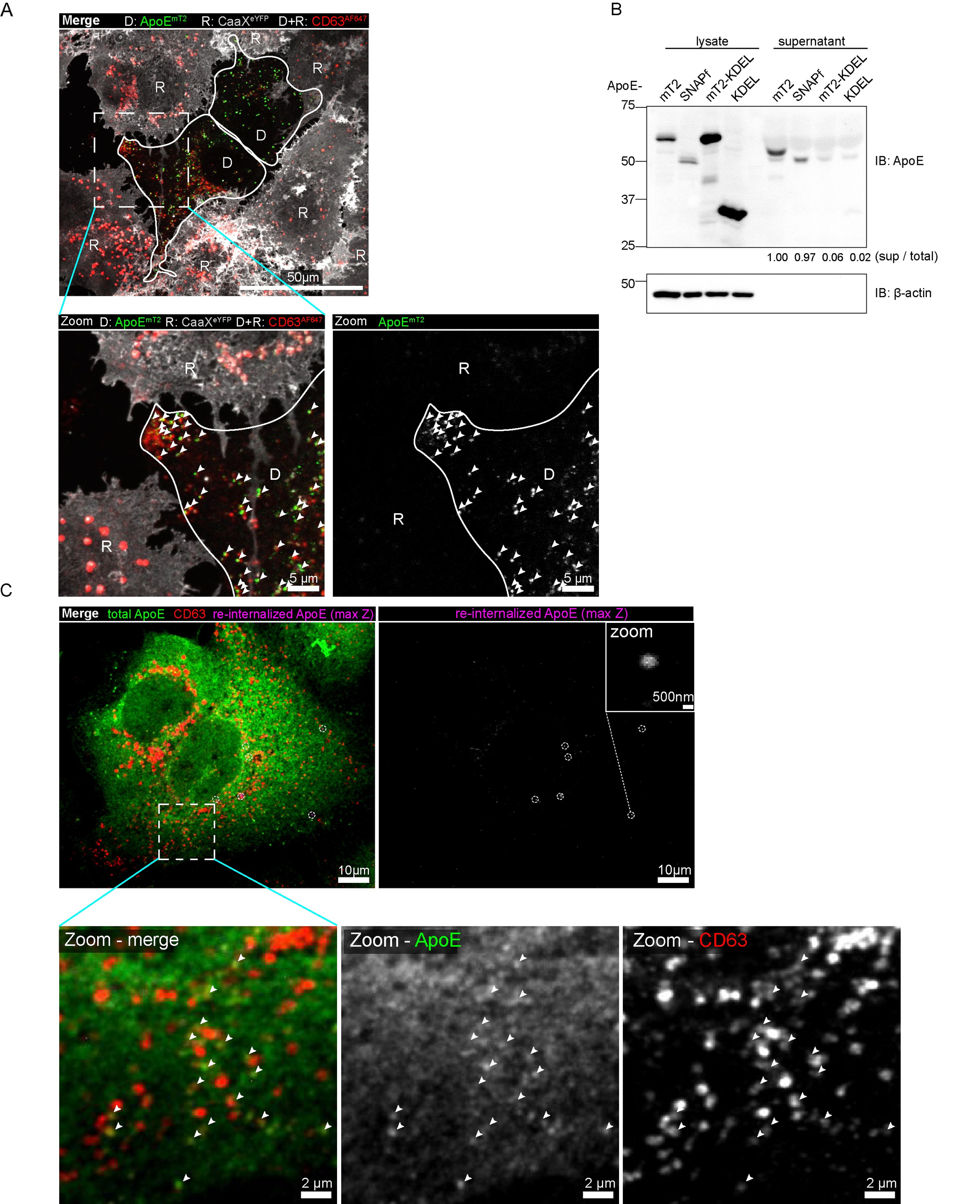

Supplement: S5 Fig — (A) Huh7-Lunet/ApoE-KD cells were lentivirally transduced with the ApoEmT2 expression vector for 4 h (donor). Cells were then washed twice with PBS and Huh7-Lunet/ApoE-KD recipient cells expressing the eYFPCaaX membrane sensor were added. After 12 h, cells were fixed, subjected to immunostaining to label CD63, and analyzed by confocal microscopy. Boxed area in the top panel is shown as an enlarged view in the panels on the bottom. Arrowheads point to newly synthesized ApoE-CD63 double-positive signals in the donor cell. D: donor, R: recipient. (B) Expression and secretion of mTurquoise2-, SNAPf- and KDEL-tagged ApoE. Lysates and culture supernatants of Huh7-Lunet/ApoE-KD cells expressing ApoEmT2, or ApoESNAPf, or ApoEmT2-KDEL, or ApoEKDEL were analyzed by Western blot using ApoE-specific antibody. β-actin served as loading control. KDEL-tagged ApoE that is retained in the ER served as specificity control to determine ApoESNAPf secretion. The ratios of secreted to total ApoE for each given construct are displayed below the corresponding lanes. The ratio of ApoEmT2 was set to 1. (C) Huh7-Lunet/ApoE-KD cells were lentivirally transduced with the ApoESNAPf expression vector. After 4 h, cells were cultured in medium containing 5 μM cell-non-permeable SNAP-surface substrate for 12 h to selectively label ApoESNAPf present in the culture supernatant. Thereafter, cells were fixed, subjected to immunostaining to label total intracellular ApoE and CD63, and analyzed by confocal microscopy. The re-internalized ApoE signals (marked with dashed circle) are visualized via the SNAP-surface substrate and shown in a maximum intensity Z-projection (1.2 μm, 0.2 μm/step). Boxed area in the top panel is shown as an enlarged view in the panels on the bottom. Arrowheads point to newly synthesized (i.e. SNAP-substrate negative) ApoE-CD63 double-positive signals of a shown focal plane (dashed circles, upper left and right). (TIF) [file ppat.1011052.s005.tif]

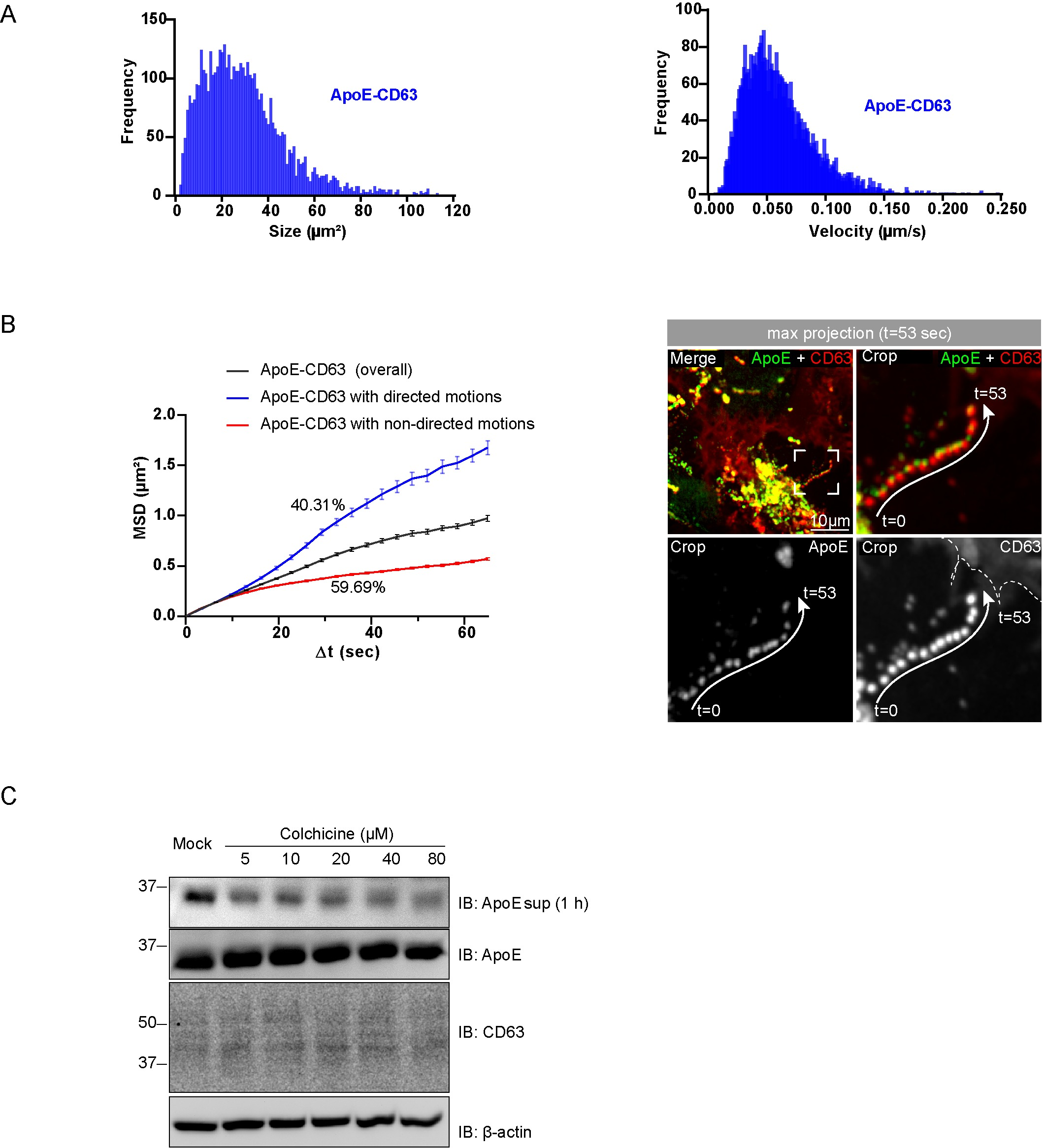

Supplement: S6 Fig — (A) Huh7-Lunet/ApoEmT2 cells expressing CD63mcherry were analyzed by live-cell confocal microscopy. Histograms of sizes of ApoE-CD63 double-positive structures (left) and their trafficking velocities are shown (right). (B) [Left] Mean squared displacement (MSD) of overall ApoE and CD63 trafficking of double-positive structures from (A) and comparison with MSD of those structures displaying directed or non-directed motions. [Right] Example of ApoE-CD63 co-trafficking by directed motion to the cell periphery. Huh7-Lunet/ApoEmT2 cells expressing CD63mcherry were analyzed by live-cell confocal microscopy. A maximum projection image showing co-trafficking of an ApoE-CD63 complex with a directed motion to the cell periphery is shown. Frame interval = 2.65 sec; whole duration = 53 sec. (C) Huh7-Lunet cells were either mock-treated or treated with increasing concentration of colchicine for 1 h to depolymerize microtubules. ApoE in cell lysates and in the supernatants was analyzed by Western blot using ApoE-specific antibody; CD63 in cell lysates was analyzed by Western blot using CD63-specific antibody. β-actin served as loading control for cell lysates. (TIF) [file ppat.1011052.s006.tif]

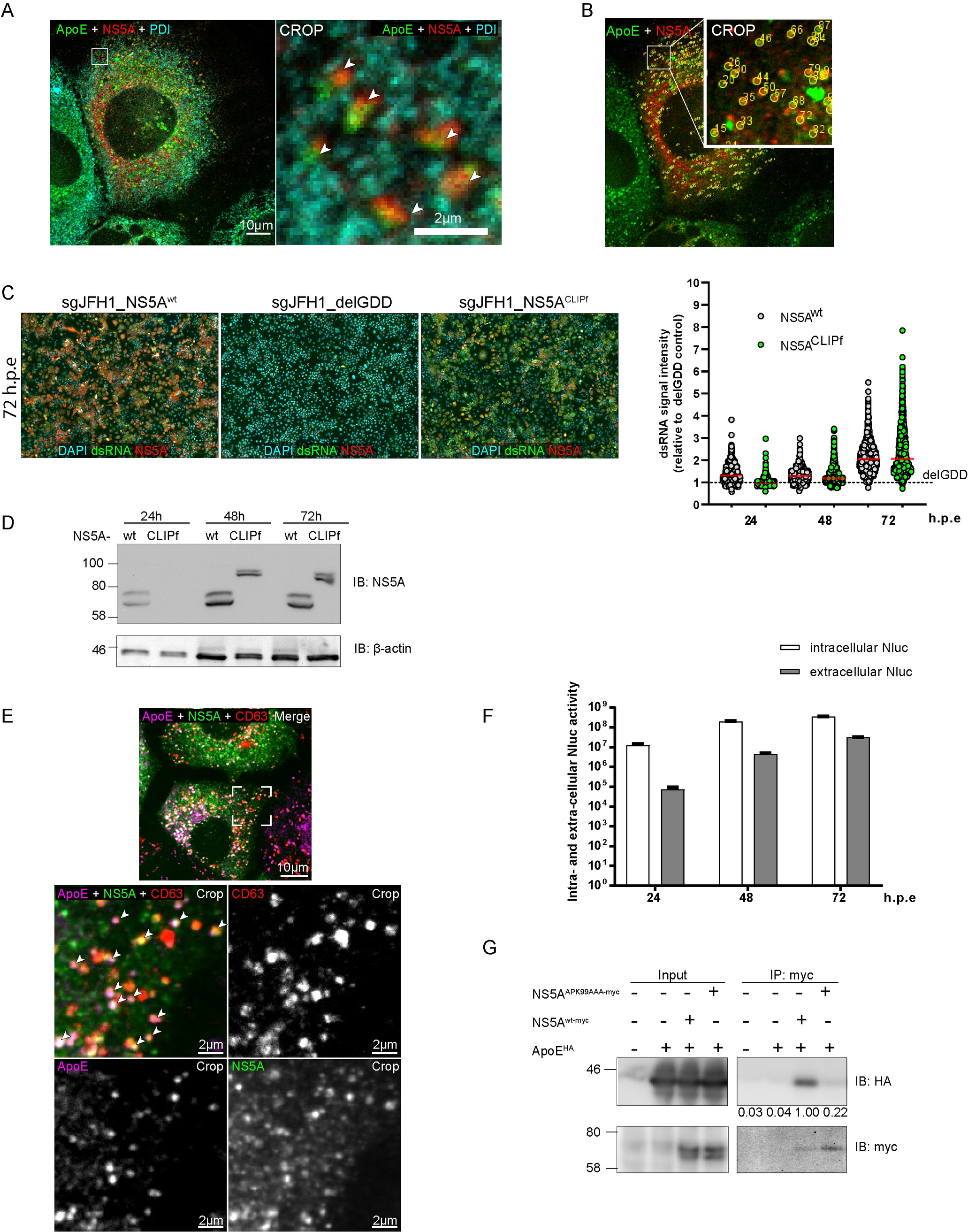

Supplement: S7 Fig — (A) ApoE-NS5A colocalization in cells replicating a full-length HCV genome. Huh7-Lunet/ApoEmT2 cells were electroporated with in vitro transcripts of the HCV genome Jc1. At 54 h post-electroporation, cells were fixed, permeabilized, and incubated with NS5A- and PDI-specific antibodies for subsequent immunofluorescence staining. Images were acquired with a confocal microscope. Arrowheads: ApoE-NS5A signals. Note the high similarity to the structures detected in cells containing the split HCV genome (Fig 5). (B) Example of automated detection and visualization of ApoE-NS5A double-positive puncta from (A) using ColocQuant and ColocJ. Circles and numbers mark the identity of each detected ApoE-NS5A double-positive structure. (C-D) CLIPf-tagged NS5A supports HCV RNA replication. (C) Detection of double-stranded RNA (dsRNA) in cells transfected with sgJFH1/NS5ACLIPf. Huh7-Lunet cells electroporated with RNA of sgJFH1/NS5Awt, or sgJFH1/NS5ACLIPf or the replication-defective mutant sgJFH1/NS5Awt/NS5BdelGDD were fixed at 24, 48, and 72 h post-electroporation (p.e.), subjected to immunofluorescent staining of dsRNA, NS5A, and nuclear DNA, and analyzed by wide-field microscopy. Quantitative analysis of dsRNA signal intensity in single cells at indicated time points after normalization to the delGDD control is shown in the right panel. (D) Expression of CLIPf-tagged NS5A. Huh7-Lunet cells were electroporated with RNA of the subgenomic replicon sgJFH1/NS5Awt or sgJFH1/NS5ACLIPf, and cell lysates harvested at 24, 48, and 72 h post-electroporation were analyzed by Western blot using NS5A-specific antibody. β-actin served as a loading control. (E) Colocalization of ApoE-NS5A double-positive structure with CD63. Huh7-Lunet/ApoESNAPf cells were electroporated with subgenomic replicon RNA encoding NS5ACLIPf and after 72 h, cells were sequentially labeled with SNAPSiR647 and CLIPATTO590 for 1 h, fixed, permeabilized, incubated with anti-CD63AF488 antibody, and subjected to confocal mic [file ppat.1011052.s007.tif]

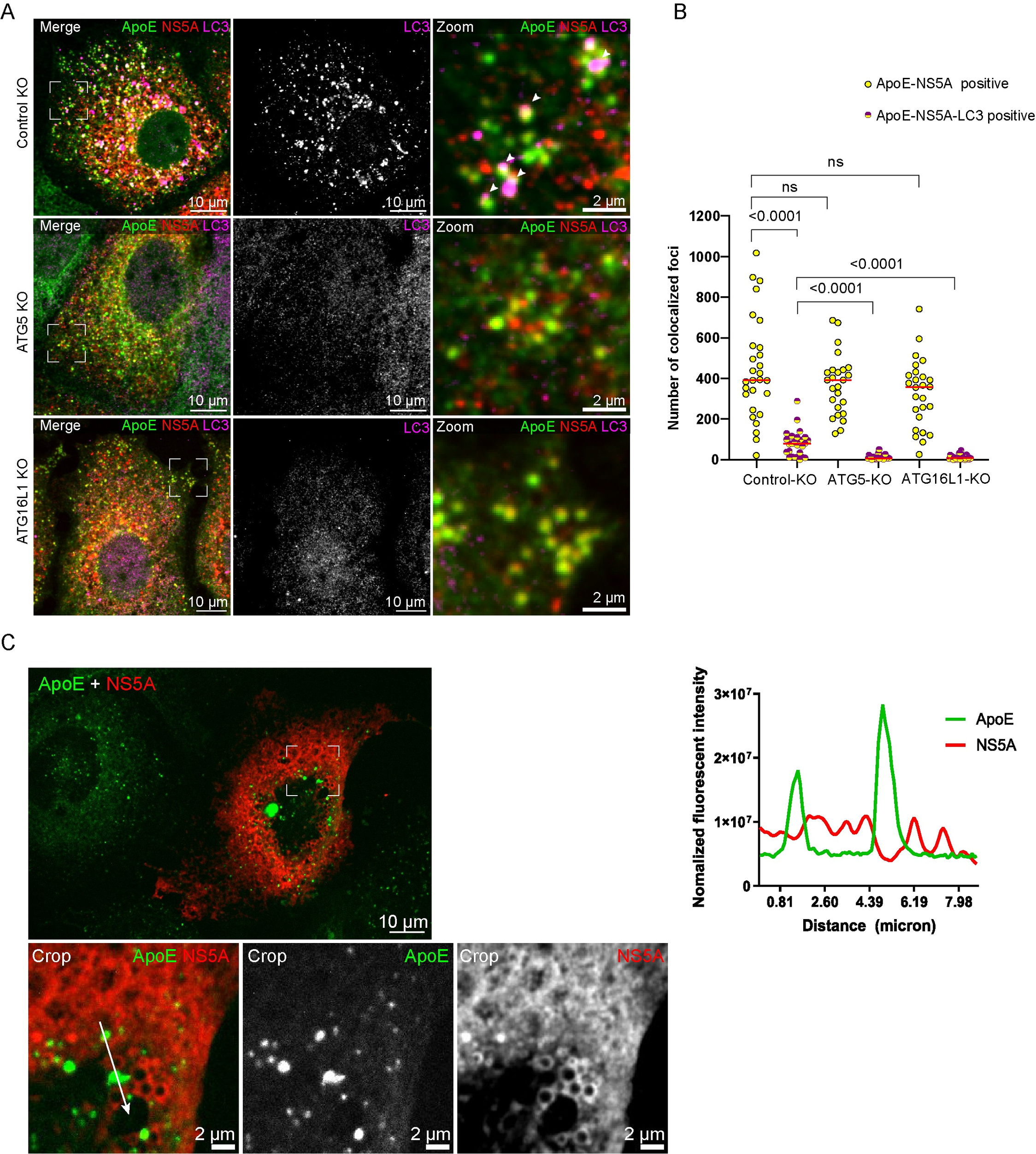

Supplement: S8 Fig — (A-B) Unaltered enrichment of NS5A in autophagy-deficient cells. (A) Huh7-Lunet cells with knock-out (KO) of ATG5 or ATG16L1 and control-KO cells were electroporated with HCV subgenomic replicon RNA encoding NS5AmCherry. After 54 h, cells were fixed and subjected to immunofluorescence staining of ApoE and LC3 and analyzed by confocal microscopy. Boxed areas in the left panels are shown as enlarged views in the panels on the right of each row. Arrowheads point to ApoE-NS5A-LC3 triple-positive signals. (B) The numbers of ApoE-NS5A double-positive and ApoE-NS5A-LC3 triple-positive signals in single cells from (A) are shown. P-values were determined using Mann-Whitney test. (C) Subcellular distribution of NS5A expressed on its own in relation to ApoE. Huh7-Lunet/ApoEmT2 cells were transfected with NS5A expression construct. Cells were fixed at 72 h post-transfection, subjected to immunofluorescence staining of NS5A, and analyzed by confocal microscopy. Boxed area in the top panel is shown as an enlarged view in the panels on the bottom. Plot profile in the right panel is along the line indicated with the white arrow in the lower left crop image. (TIF) [file ppat.1011052.s008.tif]

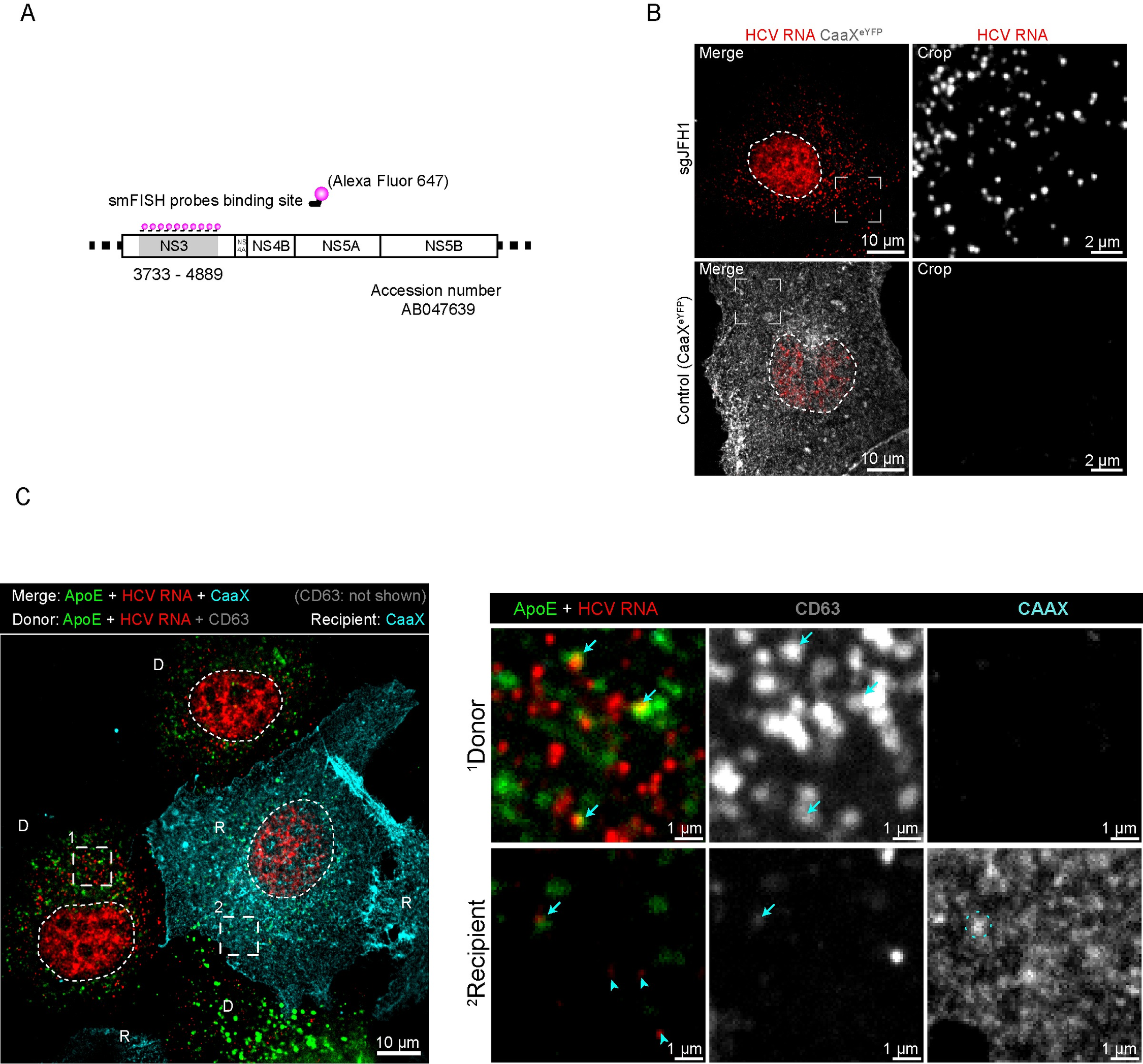

Supplement: S9 Fig — (A) Schematic of the design of smFISH Hulu probes used to detect HCV RNA. These probes target a region encoding for NS3 (nucleotide 3733–4889 of the HCV JFH1 genome; GenBank accession number AB047639). (B) Specificity of HCV RNA detection by smFISH with Hulu probes. HCV RNA contained in Huh7-Lunet cells harboring a subgenomic replicon was detected by smFISH. Huh7-Lunet cells expressing the membrane sensor eYFP-CaaX (farnesylation signal from human HRAS protein) and used as recipient cells in co-culture experiments served as a negative control. The boundaries of cell nuclei were marked with white dashed circles, and the areas within these circles were excluded from the analysis to omit unspecific staining by the RNA probes. (C) Detection of ApoE-associated HCV RNA in recipient cells. Huh7-Lunet/ApoEmT2/CD63mCherry cells containing a subgenomic HCV replicon (donor cells) were co-cultured with Huh7-LuneteYFP-CaaX recipient cells for 24 h. Thereafter, cells were fixed and processed for visualization of HCV RNA by using smFISH. An overview image is shown on the left. Dashed area 1: donor cell; dashed area 2: recipient cell. Magnified views of dashed areas are shown on the right panels. Arrows point to ApoE-associated HCV RNA dots detected in both donor and recipient cells. Arrowheads indicate ApoE-CD63 double-negative HCV RNA dots in the recipient cell. (TIF) [file ppat.1011052.s009.tif]
