# Supplementary material for: Endosomal egress and intercellular transmission of hepatic ApoE-containing lipoproteins and its exploitation by the hepatitis C virus
Source: PLoS Pathog. 2023 Jul 28;19(7):e1011052. doi: 10.1371/journal.ppat.1011052 (PMC10411793; doi:10.1371/journal.ppat.1011052)
Supplement: S1 Table — (DOCX) [file ppat.1011052.s010.docx]

S1 Table. Reagent or resource used in this study

| **Reagent or resource** | **Source (cat #)** | **Identifier** |
| --- | --- | --- |
| **Antibodies** | | |
| goat polyclonal anti-ApoE | Chemicon | AB947 |
| normal goat IgG | Santa Cruz Biotechnology | sc-2028 |
| mouse monoclonal anti-NS5A (9E10) | Gift from C. M. Rice | N/A |
| mouse monoclonal anti-CD63 (MX-49.129.5) | Santa Cruz Biotechnology | sc-5275 |
| mouse monoclonal anti-CD63 (Ts63) | ThermoFisher Scientific | 10628D |
| mouse monoclonal anti-CD63 Alexa Fluor 488 (MX-49.129.5) | Santa Cruz Biotechnology | sc-5275 |
| rabbit polyclonal anti-PDI | Sigma Aldrich | P7496 |
| rabbit monoclonal anti-GM130 (D6B1) XP | Cell Signalling | 12480S |
| rabbit polyclonal anti-ApoB | Abcam | ab20737 |
| rabbit polyclonal anti c-Myc | Santa Cruz Biotechnology | sc-789 |
| mouse monoclonal anti c-Myc (9E10) | ThermoFisher Scientific | MA1-980 |
| rabbit polyclonal anti-HA | ThermoFisher Scientific | PA1-985 |
| mouse monoclonal anti-HA (HA-7) | Sigma Aldrich | H3663 |
| mouse monoclonal anti-β-actin | Sigma Aldrich | A5441 |
| mouse monoclonal anti-α-tubulin | Hoelzel-biotech | A01410 |
| rabbit polyclonal anti-goat HRP | Sigma Aldrich | A5420 |
| mouse monoclonal anti-dsRNA J2 | Jena Bioscience | RNT-SCI-10010200 |
| goat polyclonal anti-mouse HRP | Sigma Aldrich | A0168 |
| goat polyclonal anti-rabbit HRP | Sigma Aldrich | A0545 |
| Alexa Fluor 488, donkey anti-goat | ThermoFisher Scientific | A-11055 |
| Alexa Fluor 568, donkey anti-mouse | ThermoFisher Scientific | A10037 |
| Alexa Fluor 568, donkey anti-goat | ThermoFisher Scientific | A-11057 |
| Alexa Fluor 647, donkey anti-goat | ThermoFisher Scientific | A21447 |
| Alexa Fluor 647 donkey anti-rabbit | ThermoFisher Scientific | A-31573 |
| Alexa Fluor 647 donkey anti-mouse | ThermoFisher Scientific | A-31571 |
| rabbit anti-goat | Dianova | 305-001-003 |
| rabbit anti-mouse | Rockland | 800-656-7625 |
| protein A coupled to 10-nm gold particles | Cell Microscopy Center, Utrecht, The Netherlands | N/A |
| **DNA plasmids** | | |
| pWPI-ApoE3 | [1] | N/A |
| pWPI-ApoE3^mTurquoise2^ | This study | N/A |
| pWPI_ApoE^SNAPf^ | This study | N/A |
| pWPI-ApoE3^mCherry^ | This study | N/A |
| pWPI-ApoE3^mScarlet-C1^ | This study | N/A |
| pWPI-ApoE3^mScarlet-H^ | This study | N/A |
| pWPI-ApoE3^mKate2^ | This study | N/A |
| pWPI-ApoE3^Taq-RFP^ | This study | N/A |
| pWPI-ApoE3^eYFP^ | This study | N/A |
| pWPI-CD63^mCherry^ | This study | N/A |
| pWPI-CD63^Nluc^ | This study | N/A |
| pWPI_eYFP-CAAX | This study | N/A |
| pFK_Jc1 | [2] | N/A |
| pFK_JcR2a | [3] | N/A |
| pFK_I389neoNS3-3′_dg_JFH1_NS5A-aa2359_mCherry_NS3-K1402Q (sgNeo/JFH1/NS5A^mcherry^) | [4] | N/A |
| pFK_I389neoNS3-3′_dg_JFH1_NS5A-aa2359_CLIPf_NS3-K1402Q  (sgNeo/JFH1/NS5A^CLIPf^) | This study | N/A |
| pFK_I389HygNS3-3′_dg_JFH1_NS3-K1402Q  (sgHyg/JFH1) | This study | N/A |
| pFK_I389HygNS3-3′_dg_JFH1_NS5A-aa2359_APK99AAA_NS3-K1402Q (sgHyg/JFH1/NS5A^APK99AAA^) | This study | N/A |
| pFK_I389HygNS3-3′_dg_JFH1_NS5A-aa2359-NLuc-NS3-K1402Q  (sgHyg/JFH1/NS5A^Nluc^) | This study | N/A |
| pCDNA3^+^ NS5A-myc | This study | N/A |
| pCDNA3^+^ NS5A-APK99AAA-myc | This study | N/A |
| pWPI-Core-NS2/E2^eYFP^ | This study | N/A |
| pWPI-Core-p7/E2^eYFP^ | This study | N/A |
| pWPI-CD63_M153R-pHluorin | This study | N/A |
| **Experimental models: cell lines/pools** | | |
| HEK293T | [5] | RRID:CVCL_0063 |
| HEK293T-miR122 | [6] | N/A |
| HEK293T-miR122/empty_vector | This study | N/A |
| HEK293T-miR122/ApoE^wt^ | This study | N/A |
| HEK293T-miR122/ApoE^mT2^ | This study | N/A |
| Huh7.5 | [7] | RRID:CVCL_7927 |
| Hela Kyoto | N/A | N/A |
| Huh7-Lunet/CD81H | [8] | N/A |
| Huh7-Lunet/CD81H/ApoE-KD | [9] | N/A |
| Huh7-Lunet/CD81H/ApoE-KD/ApoE^mT2^ (Huh7-Lunet/ApoE^mT2^) | This study | N/A |
| Huh7-LunetCD81H/ApoE-KD/ApoE^SNAPf^ (Huh7-Lunet/ApoE^SNAPf^) | This study | N/A |
| Huh7-Lunet/ApoE^mT2^/Core-NS2/E2^eYFP^ | This study | N/A |
| Huh7-Lunet/ApoE^mT2^/Core-p7/E2^eYFP^ | This study | N/A |
| Huh7-Lunet/ApoE^mT2^/CD63^mCherry^ (HCV negative donor) | This study | N/A |
| Huh7-Lunet/ApoE^mT2^/CD63^mCherry^/sgNeo-JFH1 (sgHCV donor) | This study |  |
| Huh7-LunetCD81H/^eYFP-CaaX^ (recipient) | This study | N/A |
| **RT-qPCR probes and primers** | | |
| HCV probe: 5’ - 6-FAM - AAA GGA CCC AGT CTT CCC GGC AAT T - TAMRA | Merck, Darmstadt, Germany | N/A |
| HCV qPCR primer (sense) S-146: 5’-TCT GCG GAA CCG GTG AGT A-3’ | Merck, Darmstadt, Germany | N/A |
| HCV qPCR primer (antisense) A-219: 5’-GGG CAT AGA GTG GGT TTA TCC A-3’ | Merck, Darmstadt, Germany | N/A |
| GAPDH probe: 5´- 6-VIC -CAA GCT TCC CGT TCT CAG CCT- TAMRA | Merck, Darmstadt, Germany | N/A |
| GAPDH primer (sense) S-GAPDH: 5´-GAA GGT GAA GGT CGG AGT C-3´ | Merck, Darmstadt, Germany | N/A |
| GAPDH primer (antisense) A-GAPDH: 5´-GAA GAT GGT GAT GGG ATT TC-3´ | Merck, Darmstadt, Germany | N/A |
| **Reagents and kits** | | |
| TransIT-LT1 Transfection Reagent | Mirus Biology | MIR2306 |
| NucleoSpin RNA extraction kit | Machery-Nagel | 740955 |
| HCS LipidTOX Deep Red neutral lipid stain | ThermoFisher Scientific | H34477 |
| Optiprep | Sigma Aldrich | D1556 |
| Benzonase Nuclease | EMD-Millipore | 70664 |
| Nano-Glo Luciferase Assay System | Promega | N1110 |
| Coelenterazine | PJKBiotech | 102172 |
| Hulu probe | PixelBiotech | N/A |
| ProLong Gold Antifade Mountant | ThermoFisher Scientific | N/A |
| SNAP-Cell 647-SiR | New England BioLabs | S9102S |
| CLIP-ATTO590 | N/A | N/A |
| Colchicine | Sigma Aldrich | C9754 |
| **Software** | | |
| FIJI | open-source | https://imagej.nih.gov/ij/ |
| GraphPad Prism 8.0 | LaJolla, CA, USA N/A | N/A |
| ColocQuant and ColocJ | Biomedical Computer Vision (BMCV) group, Heidelberg University | IEEE Access, doi: 10.1109/ACCESS.2023.3276232. |
| Huygens Professional version s19.10 | Scientific Volume Imaging, The Netherlands | http://svi.nl |
| Ilastik | ilastik: interactive machine learning for (bio)image analysis | https://doi.org/10.1038/s41592-019-0582-9 |
| CellProfiler | Broad Institute, Inc. | https://doi.org/10.1186/gb-2006-7-10-r100 |

**SI References**

1. Long G, Hiet MS, Windisch MP, Lee JY, Lohmann V, Bartenschlager R. Mouse hepatic cells support assembly of infectious hepatitis C virus particles. Gastroenterology. 2011;141(3):1057-66. Epub 2011/06/28. doi: 10.1053/j.gastro.2011.06.010. PubMed PMID: 21699799.

2. Pietschmann T, Kaul A, Koutsoudakis G, Shavinskaya A, Kallis S, Steinmann E, et al. Construction and characterization of infectious intragenotypic and intergenotypic hepatitis C virus chimeras. 2006;103(19):7408-13. doi: 10.1073/pnas.0504877103 %J Proceedings of the National Academy of Sciences.

3. Reiss S, Rebhan I, Backes P, Romero-Brey I, Erfle H, Matula P, et al. Recruitment and activation of a lipid kinase by hepatitis C virus NS5A is essential for integrity of the membranous replication compartment. Cell Host Microbe. 2011;9(1):32-45. doi: 10.1016/j.chom.2010.12.002. PubMed PMID: 21238945.

4. Ruggieri A, Dazert E, Metz P, Hofmann S, Bergeest JP, Mazur J, et al. Dynamic oscillation of translation and stress granule formation mark the cellular response to virus infection. Cell Host Microbe. 2012;12(1):71-85. Epub 2012/07/24. doi: 10.1016/j.chom.2012.05.013. PubMed PMID: 22817989; PubMed Central PMCID: PMCPMC3873964.

5. Graham FL, Smiley J, Russell WC, Nairn R. Characteristics of a human cell line transformed by DNA from human adenovirus type 5. J Gen Virol. 1977;36(1):59-74. Epub 1977/07/01. doi: 10.1099/0022-1317-36-1-59. PubMed PMID: 886304.

6. Hueging K, Doepke M, Vieyres G, Bankwitz D, Frentzen A, Doerrbecker J, et al. Apolipoprotein E codetermines tissue tropism of hepatitis C virus and is crucial for viral cell-to-cell transmission by contributing to a postenvelopment step of assembly. J Virol. 2014;88(3):1433-46. Epub 2013/11/01. doi: 10.1128/jvi.01815-13. PubMed PMID: 24173232; PubMed Central PMCID: PMCPMC3911621.

7. Blight KJ, McKeating JA, Rice CM. Highly permissive cell lines for subgenomic and genomic hepatitis C virus RNA replication. J Virol. 2002;76(24):13001-14. Epub 2002/11/20. doi: 10.1128/jvi.76.24.13001-13014.2002. PubMed PMID: 12438626; PubMed Central PMCID: PMCPMC136668.

8. Koutsoudakis G, Kaul A, Steinmann E, Kallis S, Lohmann V, Pietschmann T, et al. Characterization of the early steps of hepatitis C virus infection by using luciferase reporter viruses. J Virol. 2006;80(11):5308-20. Epub 2006/05/16. doi: 10.1128/jvi.02460-05. PubMed PMID: 16699011; PubMed Central PMCID: PMCPMC1472176.

9. Lee J-Y, Acosta EG, Stoeck IK, Long G, Hiet M-S, Mueller B, et al. Apolipoprotein E likely contributes to a maturation step of infectious hepatitis C virus particles and interacts with viral envelope glycoproteins. Journal of virology. 2014;88(21):12422-37. Epub 2014/08/13. doi: 10.1128/JVI.01660-14. PubMed PMID: 25122793.
