# Supplementary material for: Endosomal egress and intercellular transmission of hepatic ApoE-containing lipoproteins and its exploitation by the hepatitis C virus
Source: PLoS Pathog. 2023 Jul 28;19(7):e1011052. doi: 10.1371/journal.ppat.1011052 (PMC10411793; doi:10.1371/journal.ppat.1011052)
Supplement: Attachment — Submitted filename: Rebuttal_PlosPath_Pham.pdf [file ppat.1011052.s017.pdf]

Reviewer's Responses to Questions

We thank the editor and the reviewers for their supportive and constructive comments. We have addressed all the comments, including the editor's request to confirm that overexpressed tagged ApoE faithfully recapitulates the behavior of the endogenous protein and that double ApoE-CD63 positive structures in the recipient cells may form due to independent secretion and endocytosis events. Below please find our item-by-item responses, each highlighted in blue.

Reviewer #1: In this paper, the authors analyzed the egress of apolipoprotein E (ApoE) and its potential exploitation by hepatitis C virus (HCV). ApoE is synthesized at the endoplasmic reticulum and transported to the Golgi where it associates with lipoproteins. The authors focused their work on the transport of ApoE-lipoproteins between the Golgi and the plasma membrane. For this, they developed a fully functional fluorescently tagged ApoE which allows to follow the trafficking and egress of ApoE in hepatocytic cells. They showed that ApoE-lipoproteins accumulate in CD63 positive endosomes. Their data suggest co-secretion and cell-to-cell co-transmission of ApoE and endosome-derived extracellular vesicles. They also showed intracellular enrichment of HCV NS5A in ApoE-positive structures. They also observed the presence of NS5A- and ApoE-containing intraluminal vesicles in endosomes. Finally, their data suggest co-secretion and co-transmission of ApoE-positive lipoproteins with endosome-derived extracellular vesicles containing HCV NS5A and viral RNA. Globally, this work is technically sound. However, it remains rather descriptive. In particular, the localization of proteins in late endosomes does not necessarily mean that they are transported from the Golgi to this compartment. Indeed, large amounts of material ends up in late endosomes after being taken up from the extracellular medium or by autophagy. It is therefore needed to exclude these hypotheses to confirm the conclusions.

<u>Major comments</u>

1. In Huh-7 cells, apoE-mT2 is observed as dots by immunofluorescence, which are localized in the secretory (PDI, GM130) and endocytic (CD63, rab7) pathways. However, it is not clearly demonstrated where the endosomal apoE-mT2 originates from. Is it transported from the Golgi to endosomes, or is it secreted in the medium and then re-internalized by endocytosis to reach endosomes?

We appreciate the question raised by reviewer and performed additional experiments. Obtained results corroborate that newly synthesized ApoE-CD63 co-labeled structures correspond to ApoE targeted to CD63-positve endosomes in donor cells, rather than to ApoE re-internalized from the extracellular medium. The results are shown in the new Figure 2C. The revised manuscript reads as follows: "To exclude the possibility that detected ApoE foci in late endosomes corresponded to secreted ApoE that was re-internalized into cells by endocytosis, we compared the number of endocytosed ApoE-containing structures, relative to total intracellular, newly synthesized ApoE structures. To this end, we cocultured freshly transduced $ApoE^{mT2}$ cells (donor) with excess number of bystander (recipient) cells expressing the human HRAS-derived CaaX peptide that was fused to eYFP for 12 h (Fig 2C, upper left panel). The CaaX motif, which is a farnesylation signal from the human HRAS protein, targets the eYFP fusion protein to cellular membranes making them easily trackable via eYFP and allowing the faithful discrimination of recipient and donor cells. In this setting, donor cell-transmitted ApoE detected in recipient cells corresponds to secreted and re-internalized ApoE. Using this approach, we observed in donor cells newly expressed ApoE forming numerous dotted structures colocalizing with about a quarter of total detected CD63 signals in these cells (Figs 2C, panel i. and S5A). Importantly, the number of re-internalized ApoE detected in $CaaX^{eYYFP}$ recipient cells was very low (Fig

2C, panel i.). As second approach, we generated an expression construct encoding a functional SNAP-tagged ApoE^{SNAPf} (S5B Fig). In this case, cells expressing ApoE^{SNAPf} were cultured in medium containing the cell-nonpermeable SNAPf fluorophore for 12 h to label secreted ApoE and track its presence in cells after re-internalization (Fig 2C, lower left panel). Also in this setting, re-internalization of ApoE was negligible compared to the overall high number of ApoE-CD63 double-positive foci (Figs 2C, panel ii. and S5C). These results suggested that newly synthesized ApoE-CD63 co-labeled structures corresponded to ApoE targeted to CD63-positve endosomes in donor cells, but not to ApoE re-internalized from the extracellular medium."

2. What is the intracellular localization of apoE-mT2 in HeLa and 293T cells? Secretory or endocytotic pathways or both, like in Huh-7 cells? Is it secreted associated to lipoproteins in these non-hepatic cell lines, which do not express apoB?

To answer this question, we examined the intracellular localization of ApoE^{mT2} in Hela and HEK293T cells. Based on obtained results (new S4 Fig), we modified the manuscript as follows: "Evidence for localization of ApoE to the secretory and the endosomal pathway was also obtained with the non-hepatic cell lines HEK293T and Hela that support HCV particle production upon ectopic ApoE expression [40]. When we expressed ApoE^{mT2} in these cells, we observed its colocalization with both endosomal (PDI, GM130) and endocytic (CD63, Rab7) markers (S4 Fig), suggesting that also in these non-hepatic cells ApoE traffics via an endosomal pathway.", similar to what we found for Huh-7 cells.

Related to the remaining questions, we can only speculate whether ApoE secreted from Hela or HEK293T cells is associated with lipoprotein but want to point out that ApoE has been demonstrated to associate to lipoproteins as the sole apolipoprotein, i.e. in absence of ApoB (PMID: 16452453). We elaborate on this aspect in the first paragraph of the discussion.

3. It would be interesting to analyze the intracellular localization of apoE-mT2 in cells which do not internalize lipoproteins by endocytosis.

We agree it would be interesting to study this aspect, but we are not aware of cells that do not internalize lipoproteins by endocytosis. In fact, uptake of lipoprotein into cells appears to be mediated by multiple pathways, many of them still poorly defined. Therefore, inhibition of this process is difficult and obtained results will be flawed. Nevertheless, the two experimental approaches described in our reply to item #1 of this reviewer address this aspect at least in part.

4. Fig 3, the argument of co-secretion and co-transmission is rather weak. Both structures could very well be secreted independently and associate in the extracellular medium or in the endocytic pathway of recipient cells. The authors should quantify ApoE+CD63- spots and apoE-CD63+ spots in recipient cells in addition to double positive spots. In addition, it would be interesting to study the effect of exosome secretion inhibitors. Would they inhibit ApoE and CD63 release to the same extent?

We totally agree with the concern raised by the reviewer. We now provide the quantification of ApoE^{+}CD63^{-} spots and ApoE^{-}CD63^{+} spots in recipient cells in the new Fig 4E. In addition, we toned down the statement of "co-secretion" and "co-transmission" accordingly in the entire manuscript, including our discussion that now reads: "Our data suggest that in naïve and HCV-infected hepatocytes, ApoE-LPs and endosome-derived CD63-positive ILVs/EVs share a common intracellular late endosomal trafficking in a microtubule dependent and autophagy independent manner. In addition, they appear to be co-secreted and internalized into bystander cells. Although we cannot rule out that these two particle species are separately secreted and associate at later stages in the extracellular milieu or

during endocytosis, we postulate that at least a fraction of ApoE-CD63 double-positive structures form intracellularly and are co-secreted. This assumption is based on the observation that microtubule depolymerization concomitantly reduced the deposition of ApoE-CD63 double-positive complexes at the cell periphery and mitigated their secretion, implying that ApoE- and CD63-containing structures within endosomes might be co-secreted. This is in line with our finding using the $CD63^{pHluorin}$ construct showing the secretion of ApoE-containing CD63-positive ILVs. While further work will be needed to provide more direct evidence for this hypothesis, our data argue for a stable interaction between ApoE-LPs and CD63-positive ILVs/EVs."

Re. to exosome secretion inhibitors to examine the secretion of ApoE and CD63, several studies reported lack of inhibition of $CD63^{+}$ EVs by using numerous types of inhibitors, targeting ion balance, lysosomes, cytoskeleton, lipid pathways (GW4869, methyl β-cyclodextrin…) and others (PMID: 31339911; PMID: 34282141, PMID: 26387950). Nevertheless, to address this point of the reviewer (and item #2 of reviewer #2), we employed a microtubule-depolymerizing agent (colchicine) and included obtained data in the main manuscript as follows: "To further dissect the possible involvement of the microtubule network in the trafficking of ApoE-CD63-positive endosomes in hepatocytes, we treated Huh7-Lunet/$ApoE^{mT2}$/$CD63^{mCherry}$ cells with colchicine to depolymerize microtubules [28]. As expected, cells showed significant abrogation of ApoE and CD63 motility as deduced by their smaller MSD values over time (S2 Movie, Fig 3A, dotted lines). In addition, quantitative analysis revealed no major differences between the sizes of ApoE-CD63 double-positive particles in mock and colchicine-treated conditions (Fig 3B), but their velocities were dramatically reduced in the latter case (Fig 3C). Interestingly, in mock-treated condition, we frequently observed a large population of ApoE-CD63 double-positive particles at the cell periphery (Fig 3D, upper panels, arrows), but this population was profoundly reduced upon colchicine treatment (Fig 3D, lower panels). Quantitatively, radial CD63 signal intensity in the cell periphery of colchicine-treated cells decreased significantly (Fig 3E), suggesting that the intact microtubule network is required for the transport of both total $CD63^{+}$ and ApoE-containing $CD63^{+}$ endosomes to the cell periphery. Consistently, Huh7-Lunet cells treated with various concentrations of colchicine (5 - 80 µM) for 1 h released significantly less ApoE into the cell culture supernatant as determined by Western blot (S6C Fig). In addition, we employed Nanoluciferase (Nluc)-tagged CD63 (CD63-Nluc)-expressing cells to allow the rapid and sensitive measurement of CD63 secretion [50-52], along with the quantification of ApoE secretion in mock-and colchicine-treated cells by Elisa. We observed a parallel reduction of ApoE and CD63 secretion (Fig 3F and 3G). Taken together, our data argue for a proportion of intracellular ApoE egressing via the CD63 endosomal pathway in a microtubule-dependent manner."

5. Fig 6, the NS5A-ApoE spots appear associated to double membrane vesicles in EM. Could these vesicles be replication complexes addressed to endosomes by autophagy? Would NS5A be directed to endosomes in autophagy-deficient cells? What about NS5A expressed alone? Does it co-localize with ApoE in CD63-positive endosomes?

To answer these questions, we examined the subcellular localization of ApoE in relation to LC3 puncta, which are markers of autophagosomes. Obtained results are incorporated in our revised main text as follows: 1) "Given the important role of autophagy in the degradation of intracellular content, including material in late endosomes, we wondered whether ApoE resides in CD63-positive late endosomes of hepatocytes as result of autophagy. However, only a minor fraction of $ApoE^{mT2}$ colocalized with LC3 puncta suggesting that autophagy is not required for the enrichment of ApoE in late endosomes (Fig 2B)." and 2) "To determine whether the enrichment of NS5A in ApoE-containing endosomes is autophagy-dependent, we employed autophagy-deficient Huh7-derived cells with stable knockout (KO) of ATG5 and ATG16L1 [70]. We observed a comparatively high number of ApoE foci in these KO cells and KO-control cells suggesting that autophagy is not required for the sorting of ApoE in late endosomes (S8A and S8B Fig), which is consistent with our previous observation (Fig 2B).

Moreover, enrichment of NS5A in ApoE-containing endosomes is not strictly autophagy-dependent, because the numbers of ApoE-NS5A double-positive foci were comparable in autophagy-deficient and KO-control cells (S8A and S8B Fig). Interestingly, a minor fraction of these foci colocalized with LC3 puncta in KO-control cells, but not in autophagy-deficient cells (S8A, arrowheads and S8B Fig), suggesting that ApoE-NS5A double-positive endosomes might have fused with autophagosomes. Of note, we did not readily observe enrichment of NS5A in ApoE^{+} foci in the context of single NS5A protein expression; instead, under those conditions NS5A signals appeared to localize in ApoE-devoid areas (S8C Fig)."

Regarding the question "could these vesicles be replication complexes addressed to endosomes by autophagy?", we consider it unlikely that canonical autophagy plays a major role in this process because autophagy is dispensable for the enrichment of both ApoE and NS5A in these organelles. A more comprehensive study is needed to address this question and therefore, we did not elaborate further in our revised manuscript.

Reviewer #2: In this manuscript, Pham et al. aim at characterizing the intracellular trafficking of ApoE and understanding how ApoE egress is hijacked by HCV. To achieve these goals, the authors designed a fully functional FP-tagged ApoE and employed an integrative imaging approach to monitor the endosomal post-Golgi trafficking egress and transmission route of hepatic ApoE-LPs in uninfected cells and in the context of HCV infection.

The manuscript contributes to understanding the role of ApoE in cell biology and in viral infection. Moreover, the newly designed fusion protein represents an improved tool for tracking ApoE and monitoring its trafficking in normal and disease states. The novelty of the work is the discovery that ApoE-LPs co-egress with CD63-positive extracellular vesicles (EVs) via late endosomal trafficking and are transmitted from cell to cell. Moreover, HCV NS5A binding to ApoE is required for the release of these ApoE-associated CD63-positive EVs containing viral RNA that are transmitted between cells. Adequate controls were used and the conclusions are supported by the data.

Nevertheless, a few experiments/analyses are proposed to strengthen the conclusions, and major editing is required to improve the clarity.

Major comments:
1. Figure 1. a. There is no definition of naïve control – is this mock transfection?

Naïve cells are the unmodified parental (Huh7-Lunet) cells. Endogenous ApoE expression of the naïve cells was stably depleted to undetectable level and subsequently reconstituted with ApoE-FPs (Huh7-Lunet/ApoE-KD/ApoE-FPs). We extended our explanation in the corresponding figure caption.

b. In 1C and some of the other figures (e.g. 2A) the y-axis title says "normalized fluorescence intensity", however, it seems that raw values are plotted. If indeed normalized, what is it normalized to?

Indeed, raw values of one selected channel were kept unchanged. The values of the other channels along the same line were normalized by multiplying them with the quotient of the mean intensity of the selected channel to that of the remaining channels. Because fluorescence intensity in different channels varies, it is necessary to have them normalized for the plot profiles. We added an explanation within the "Immunofluorescence staining and confocal microscopy" section of the Materials and Methods.

c. Figure 1D – How do the authors explain the finding that mT2-ApoE rescued the various phenotypes more than WT ApoE? The expression levels of the two proteins should be shown.

Rescue by $ApoE^{mT2}$ was a bit higher, but in 2 out of 3 cases statistically insignificant (see Figure 1D). The expression levels of two proteins are shown in the new S1C Fig, including a quantification of intracellular ApoE levels of these cell pools. Expression level of $ApoE^{mT2}$ is ~1.25-fold higher than $ApoE^{wt}$, which would explain the slightly higher rescue efficiency in $ApoE^{mT2}$ cells.

2. Figure 2. a. Figure 2D: An experiment showing the microtubule-dependent trafficking of Apo-CD63 endosomes would be an interesting addition to the paper.

We totally agree and conduced the suggested experiment. Obtained results have been addressed in our reply to point #4 of reviewer #1.

b. Figure 2E (upper panel) depicts a single particle. What is the abundance of the ApoECD63pHluorin+ve particles?

We now provide the quantification of ApoE-$CD63^{pHluorin}$ double-positive particles in new Fig R1. Although the pHluorin-tagged CD63 construct is good for qualitative examination of the secretion of ApoE-associated CD63-containing intraluminal vesicles, it does not reflect the total number of secreted particles. This is due to the fact that signals need to be imaged at the cover slip-cell interface, which confers a spatial hindrance for effective secretion.

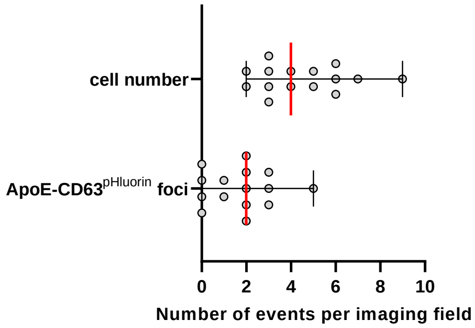

Fig R1. *Huh7-Lunet cells expressing $ApoE^{mT2}$ and $CD63^{pHluorin}$ were cultured in imaging medium (pH 7.4) and examined by live-cell confocal microscopy with a focus on plasma membrane resident CD63-fluorescent signals. Data are medians (range) of ApoE-$CD63^{pHluorin}$ foci detected in 15 imaging fields (n=68 cells).*

3. Figure 3. a. Figure 3B. The morphology of the gold-ApoE vesicles is disrupted in contrast to the CD63+ vesicles. Can the authors speculate/elaborate on what might lead to the distortion of the vesicles during gold-ApoE vs. gold-CD63 labeling?

It is well-known that apolipoproteins including ApoE play a crucial role in the stabilization of lipoprotein structure (PMID: 6099394). Addition of ApoE to lipid particles leads to the redistribution of the lipids at the shell and the core (PMID: 33754708). During the immuno-gold labeling procedure,

anti-ApoE antibody, linker antibody, and protein A-gold incubation probably interfere with ApoE association with lipoproteins, likely resulting in the leakage of lipoprotein-inner lipid core. We explain this in the revised text.

b. On a related note, only large vesicles are positive for CD63, as shown in the bottom panel. What distinguishes the larger vesicles stained positive for CD63 from the small vesicles (except size)? It would be important to determine the fraction of CD63-positive EVs in secreted ApoE.

LP and EV overlap in size, density and many other biophysical properties. The small vesicles separated in the supernatant of Huh7-Lunet cells correspond primarily to lipoproteins that lack CD63; the larger vesicles correspond predominantly to EVs containing CD63, which is a reliable and commonly used marker localizing to the EVs and discriminating EVs from LPs.

On the other hand, EVs and LPs have lipid membrane bilayer and monolayer, respectively, but this difference was not visualized by the negative staining and TEM analysis used in this study. A comprehensive investigation of the morphologies of these particles requires cryo-EM, which is beyond the scope of the present study.

We appreciate the point raised by the reviewer to determine the fraction of CD63-positive EVs in secreted ApoE. However, this is a major challenge in the field of EV research, because in *situ* measurement methods of LP-EV association in conditioned medium are not well established. Although our immuno-labeling approach of isolated ApoE-LPs provided qualitative data for stable association of LP-EV, it did not address quantitatively the degree of their association. However, we once again validated the presence of CD63 in ApoE-enriched fractions using cells expressing Nluc-tagged CD63. Our results confirmed the genuine association of a subset of secreted ApoE-and CD63-containing structures, which can withstand the precipitation process (Fig R2).

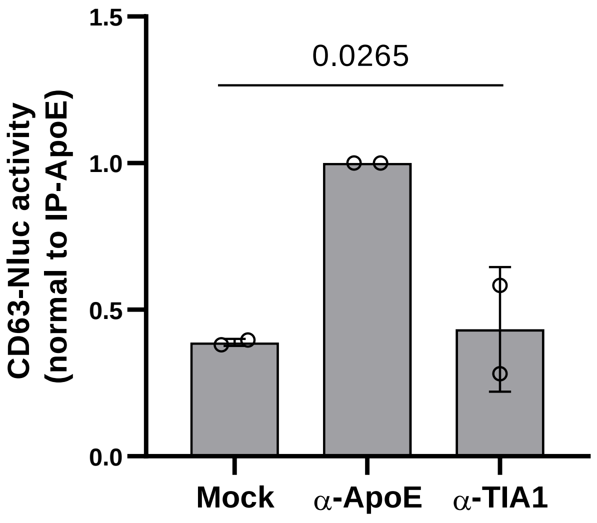

Fig R2. *Culture supernatants of Huh7-Lunet/CD63^{Nluc} cells were subjected to immunoprecipitation using ApoE-specific or mock or unrelated control antibody. The presence of CD63 in immuno-complexes was quantified by Nluc activity assay. Data are means (SD) from 2 independent experiments. P-value was determined using one-way ANOVA.*

c. Figure 3E. How were the numbers of foci quantified? Using what software? Further details should be provided.

The number of foci in recipient cells was measured by ColocQuant and ColocJ software suite, which is suitable for colocalization analysis of spot-like objects in multi-channel fluorescence microscopy images. We have published this software suite very recently:

C. Ritter, R. Thielemann, J. -Y. Lee, M. T. Pham, R. Bartenschlager and K. Rohr, "Multi-Channel Colocalization Analysis and Visualization of Viral Proteins in Fluorescence Microscopy Images," in IEEE Access*, vol. 11, pp. 49772-49784, 2023, doi: 10.1109/ACCESS.2023.3276232.*

We cited this reference in our revised manuscript.

4. Figure 4. Line 341-342: "In agreement with a previous report (60), we observed time-dependent secretion of NS5ANluc into the cell culture supernatant (Fig 4F)." There is no Fig 4F in the manuscript.

We fixed this mistake; we meant Fig S4F. This is now S7F Fig in the revised manuscript.

5. Figure 7. a. Figure 7B. The Western blot of the same samples with anti-NS5A antibodies is missing and so is the immunoblot on whole cell lysate.

The amount of NS5A in the supernatant was below the detection limit by Western blot (please see for Fig R3 and R4 referring to Fig 7A and 7B). Therefore, we employed Nluc-based assay for the sensitive detection of NS5A (Fig 7B).

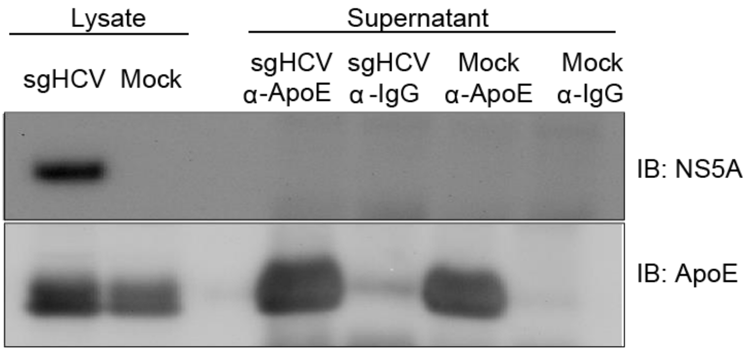

Fig R3. *Huh7 cells harboring a subgenomic HCV replicon and control cells were cultured in medium containing 1% FCS for 6 h. Culture supernatants were subjected to immunoprecipitation using ApoE-specific or IgG control antibodies. Cell lysates and immuno-captured complexes were subjected to Western blot analysis using ApoE and NS5A-specific antibodies.*

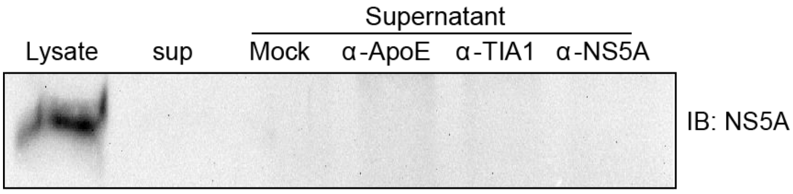

__Fig R4__. Huh7-Lunet cells were electroporated with subgenomic replicon RNA encoding the NLuc-tagged NS5A and 72 h p.e, culture supernatant was subjected to immunoprecipitation using ApoE-, or NS5A-, or control TIA1-specific antibodies. NS5A contained in cell lysate, supernatant, and immuno-captured complexes was analyzed by Western blot using NS5A-specific antibody.

b. A clearer explanation regarding the TEM findings shown in Figure 7C will help the readers.

We provide a clearer explanation in our revised main text that reads as follows: "Next, aiming to determine the direct association of HCV-produced EVs and LPs, we analyzed immunocaptured samples by negative-staining and TEM. Staining of complexes captured with NS5A- or ApoE-specific antibodies revealed EV-like structures with diameters of ~100nm that were frequently associated with LP-like particles (Fig. 8C, arrows). As expected, in ApoE-specific pulldown, LPs were more frequent, yet these samples also contained EVs associated with LPs. These results show the direct association of HCV-produced EVs with LPs."

c. Line 370-372: "Remarkably, we could detect distinct foci of HCV RNA in single recipient cells, around 13% of them being ApoE-CD63 double-positive (example image in Fig S5C, area 2; quantification in Fig 7F)." Is 13% biologically significant? What is the basal level? More details should be provided about this quantification.

The number of events was rather low as summarized in the new Fig 8F in the revised manuscript (Fig 7F in the original submission). Originally, we conducted manual counting to determine the number of events. We quantified again the data using ColocQuant and ColocJ and added more details about this quantification in the corresponding figure caption. The ratio of ApoE-CD63 positive to total HCV RNA foci remains unchanged.

Related to biological significance, we had already admitted that this is one of the limitations of our study and had explained it in the original manuscript: "The physiological consequences of co-spread of hepatic LPs with EVs in general and in the context of HCV infection, the latter possibly allowing HCV RNA spread independent of virus particles (34-37), remain to be determined but they are beyond the scope of the present study."

6. The conclusion that CD63/endosomal pathway mediate ApoE egress would be strengthened by showing that perturbation of this factor/pathway impacts ApoE egress (either in naïve of HCV-infected cells).

We appreciate the valuable suggestion of the reviewer and examined the egress of ApoE in relation to the perturbation of the CD63/endosomal pathway in naïve hepatocytes. Obtained results have been described in our reply to point #4 of reviewer #1.

<u>Minor comments:</u>

1. Materials and Methods are missing descriptions of the methods used for EV preparation and quantification of HCV core protein.

We had included this information in the original manuscript under "immunocapture of extracellular ApoE-associated structures", but following the reviewer's request, we have extended this description, including the quantification of HCV core protein (page 26, line 646-650).

2. Figure 5B (and legend): consider using consistent representation of CLIP-ATTO590 and SNAP-SIR647 in the figure and text. (For ex: In line 590, superscript in all other places).

We thank the reviewer for this comment and have formatted it accordingly in the manuscript.

3. Figure 4: Use Either h or h.p.e. in C and E for consistency.

We modified h.p.e for both Figures.

4. Line 208: "exclusively fluoresces" instead of "is exclusively excited"

We corrected the text.

5. Line 262: “spinning disc” should be spinning-disk confocal microscopy

We corrected the text.

6. Line 272: 10 sec/frame – clarify if this means each frame was taken with a 10 s exposure or whether 1 frame was taken every 10 s, in which case 1 frame/10 s may be more appropriate.

We corrected the text to read “1 frame every 10 s”.

7. Line 601: Which Alexa fluorophore?

We specified accordingly in the revised manuscript.

8. STED should be spelled out.

Done.

9. Consider rephrasing the following sentences for improved clarity:
Lines 66-69: These alterations…apolipoprotein E (ApoE).
Lines 148-150: Of note, ApoE puncta…live-cell imaging (Fig 1C).
Lines 331-333: In the first set of experiments...ApoE-specific pull-down.
Lines 403-405: For the poliovirus…apply to NS5A (73-75).

We rephrased all the mentioned sentences.

10. Figure 3D: The upper image missing a scale bar.

We added the scale bar for this figure.

Reviewer #3: The authors have developed a new tool to study ApoE secretion based on a fusion protein of ApoE with mTurquoise2. This tool allowed them to follow ApoE secretion in a hepatic cell line and to suggest that ApoE-containing lipoproteins (ApoE-LPs) follow the secretion pathway of CD63-positive late endosomes. Moreover, the authors showed that NS5A, a non-structural protein of hepatitis C virus (HCV), is enriched in ApoE-LP and follows the same late endosomal pathway for egress, and that ApoE-NS5A interaction is required for efficient release of extracellular vesicles containing the viral RNA genome. The authors have used several advanced microscopy technics such

as live imaging, super-resolution microscopy, correlative light and electron microscopy (CLEM) as well as single molecule fluorescent in situ hybridization (smFISH).
The experiments are in general well conducted, but some of them lack repetitions or controls.

Major points:
1) The assumption that over-expressed tagged proteins are fully functional is not always well shown:
a) In Fig 1B, authors should show the fractionation of a supernatant from parental Lunet cells to appreciate densities of the different over-expressed ApoE forms with regards to endogenous ApoE.

Indeed, in this figure, parental Huh7-Lunet cells were used. They are labeled as “naïve” cells, which is now better explained (see also reply to comment #1 of reviewer #2). To generate ApoE-FP expressing cells, these parental Huh7-Lunet cells were used by depleting endogenous ApoE expression to undetectable level and reconstitution with ApoE-FPs (Huh7-Lunet/ApoE-KD/ApoE-FPs).

b) In Fig S4C, authors should add ApoEmT2 to control that secretion levels between ApoESNAPf and ApoEmT2 are identical because the presented data only show that ApoESNAPf is secreted.

We provide a new blot that includes $ApoE^{mT2}$ (new S5B Fig).

c) In Fig S4D, authors should use a standard replication assay to show that NS5ACLIPf is functional for HCV genome replication.

To prove that sgHCV $NS5A^{CLIPf}$ is functional for HCV genome replication, we examined HCV double-stranded RNA (dsRNA) production over time as it is a hallmark of HCV RNA replication (PMID: 18094154). We included sgHCV NS5A/NS5B_delGDD replication-defective control. The new data are shown in S7C Fig of the revised manuscript.

2) Fig 7 and its related Fig S5 suffer from lack of repetition and controls:

a) Fig 7A: Since there are 8 dots, I assume that there are 4 replicates in each of the two experiments, which seems to indicate a high variability. Please, indicate which dots come from which experiment since otherwise, the representation of the replicate is useless and might induce misunderstanding for the readers. I would also strongly suggest increasing the number of experiments to have at least 3 independent repeats.

Also indicate which set of data was used for the statistical analysis? i.e., on internal replicates or on the mean of the 2 experiments?
What is the level of total secreted HCV RNA in these cells? The authors should add the values of non-immuno-captured HCV RNA which represents the input fraction.

We agree with the reviewer and conducted 2 more biological replicates, removed all internal technical replicates from the representation to avoid confusion and analyzed the data again based on the 3 independent biological replicates. Shown data are means (SD) of 4 biological repeats. The input RNA is also shown. This figure is now Fig 8A in the revised manuscript.

b) Fig 7B: Same as in Fig 7A, what are the values of the input fraction for NS5A (upper graph) or ApoE (lower WB)?

We included the input fraction for NS5A (upper graph) and ApoE (lower Western blot). This figure is now Fig 8B in the revised manuscript.

c) The authors should study the co-secretion of ApoE and HCV EVs from the same material. Indeed, the secretion level of HCV EVs containing HCV RNA and/or NS5A might not be the same in subgenomic HCV replicon cells (i.e., cells that stably replicate the HCV genome (Fig 7A)) than in HCV RNA transfected cells (Fig 7B).

We appreciate the point raised by the reviewer. Indeed, we were fully aware of this issue, but had to use different experimental settings for the different readouts. In Fig 7A, we employed sgHCV replicon cells to faithfully quantify HCV RNA amount in cell culture supernatant. The quantification of HCV RNA from cells transiently transfected with HCV RNA is not feasible due to the abundance of input RNA, which flaws the measurement of true secretion.

In Fig 7B, the readout is Nluc-NS5A protein amount in cell culture supernatant, which is below the detection limit by Western blot (see also our reply to comment #5 of reviewer #2). Since amounts of NS5A are below the detection limit, we had to use Nluc-NS5A as a much more sensitive read-out. However, creating stable replicon cell lines with Nluc-NS5A was not possible due to the potential rapid loss of the Nluc reporter, which would underestimate the release, and the resulting decrease in RNA replication levels caused by the reporter itself.

d) Fig 7D: I have the same concerns regarding the representation of the replicates in this panel as in Fig 7A (see above comments 2a).

We agree with the reviewer. We removed all technical replicates and analyzed the data again based on 3 independent biological repeats. Data are means (SD) of 3 biological replicates. This figure is now Fig 8D in the revised manuscript.

e) Fig S5B: Please show at least one entire cell with smFISH staining, not only a zoomed area.

We include images of entire cells (S9B Fig) in the revised manuscript.

f) Fig S5C: Please show entire cells. There is a strong red signal in the center of recipient cells: does it correspond to the background announced in line 363? If yes, how can the author discriminate background from real positive RNA signal?

We provide an image with entire cells (S9C Fig in the revised manuscript). We excluded the nuclei from the analysis because of non-specific staining by the probes within these areas. To make this clear, we marked the nuclei with dotted lines. Our analysis thus relied on subcellular localization of the signals that were outside of the nuclei. This clarification was included in the corresponding figure legend of the revised manuscript.

In line with this, HCV RNA dots in recipient cells appear to be much smaller than those in donor cells? How can the authors explain this as each single dot should represent one single RNA molecule?

This is a valid point. We note that in stable sgHCV replicon cells, HCV RNAs accumulate in the course of RNA replication. Hence, it is plausible to assume that viral RNAs accumulate in specific subcellular compartments or regions. This accumulation leads to overlapping signals in the immunofluorescence (IF) images. In contrast, given the much lower quantity of HCV RNA in recipient cells, such signal overlap would not occur, resulting in smaller dots.

We did not claim that single dots are single RNA molecules. In fact, technically, it is impossible to discriminate single particles beyond the light diffraction limit using confocal microscopy. We added a comment in the revised manuscript to make these points clear.

3) In the Materials &Methods, it is stated that cells were treated with proteinase K after permeabilization, thus, how can the authors detect mCherry, mTurquoise2 and YFP signals?

Proteinase K is used at suboptimal concentrations to improve smFISH probe signal/noise ratio while minimizing the loss of intrinsic fluorescent signals. We added a comment in the revised manuscript.

Finally, how to be sure that the ApoE-HCV RNA-CD63 dots are inside the cell and not apposed on the plasma membrane of the recipient cells?

Indeed, we cannot completely exclude that the triple-positive structures reside on the plasma membrane surface. However, at least a fraction of these structures were surrounded by the membrane marker CaaX, likely presenting endosomal compartments after endocytosis into recipient cells (dashed circle, new S9C Fig).

The authors may try to block cell entry using ApoE and/or CD63 antibody or block the exosomal secretion of donor cells with the GW4869 inhibitor and include the results in Fig 7F.

We really appreciate the idea suggested by the reviewer. However, given the low number of events and thus, very high variability even with small changes in the number of events, as well as the technical challenges of the IF-smFISH, we did not attempt to explore the suggested possibility. Additionally, as mentioned earlier in response to point #4 of reviewer #1, treatment with the GW4869 inhibitor does not significantly affect the secretion of CD63^{+}EVs (PMID: 34282141, PMID: 26387950).

<u>Minor points:</u>
1) Fig 1A-B: It is not clear whether ApoE is over-expressed in naïve cells or not. Please clarify this point in the text and in figures.

As mentioned earlier, ApoE in naïve cells is endogenous and NOT over-expressed (see also reply to comment #1 of reviewer #2).

When wt ApoE is over-expressed in naïve cells, there is a discrepancy in ApoE levels for naïve supernatants between Fig 1A and Fig 1B (the input level of ApoE in supernatant of naïve cells appears to be equal to ApoEmT2 supernatant level in Fig 1B, while there is a clear difference in Fig 1A). Which blot is representative of a standard experiment?

The blot in Fig 1A is representative of a standard experiment. We provide the same blot for Fig 1B with a shorter exposure time showing unsaturated signals and being consistent with Fig 1A.

The authors should show the parental Lunet and Lunet-ApoE KD cells to appreciate the KD efficiency of ApoE in Fig 1A.

The stable Huh7-Lunet/ApoE-KD cells were generated and carefully characterized in our former publication using various methods including qPCR, IF, and Western blot (PMID: 25122793). ApoE protein expression in these cells is not detectable.

2) In the legend of Fig S1C-D: The authors wrote in line 1310 "Data are means from a representative experiment (n=3)". Do they mean that the presented data are the means of internal replicates? Please clarify this point.

Yes, the presented data are the means of internal replicates in these Figures. We added an explanation in the legend.

3) Fig 4C: The authors mentioned in lines 270-271 that NS5AmCherry-ApoEmT2 foci abundance increased "much higher as compared to NS5AmCherry-E2eYFP positive foci", albeit they do not show quantification of the latter. Please show the quantification of NS5A-E2 positive foci. The interpretation of these quantifications should be more cautious since the HCVtcp system do not allow a huge assembly level of infectious virions, as suggested by the authors in lines 267-268.

We provide the quantification of NS5A-E2 positive foci. It is now included in Fig 5C of our revised manuscript.

We appreciate the point raised by the reviewer and mention this in the text.

4) Line 342, Fig 4F does not exist and should be replaced by Fig S4F.

We fixed this mistake; we meant Fig S4F. This is now S7F Fig in the revised manuscript.

5) Line 371-372: Do the authors mean "HCV RNA dots ApoE-CD63 double positive" or "HCV RNA ApoE positive" since in Fig 7F only quantification of HCV RNA dot that are ApoE positive is shown?

We meant ApoE-CD63 double-positive dots. For simplicity, CD63 was not indicated in this figure. We added "$CD63^{+}$" to this Figure (new Figure 8F in the revised manuscript).
